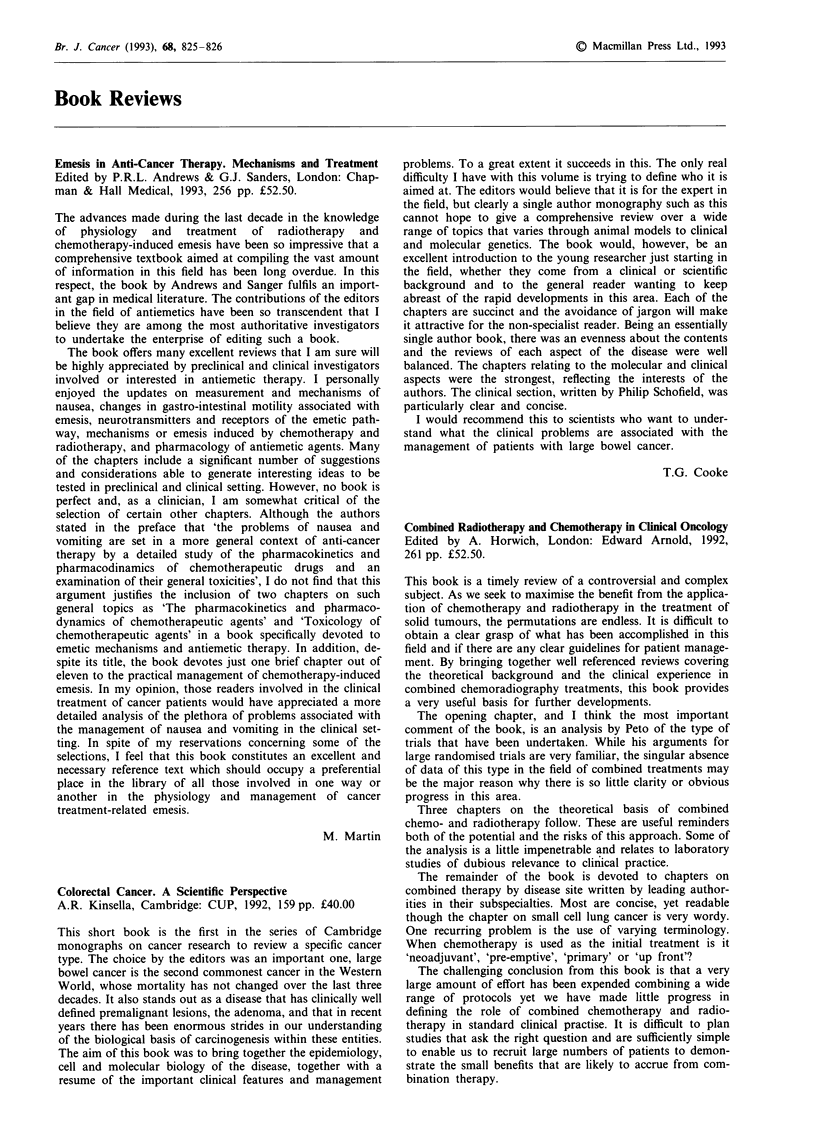# Emesis in Anti-Cancer Therapy, Mechanisms and Treatment

**Published:** 1993-10

**Authors:** M. Martin


Br. J. Cancer (1993), 68, 825–826 © Macmillan Press Ltd., 1993

### Book Reviews

**Emesis in Anti-Cancer Therapy. Mechanisms and Treatment**
Edited by P.R.L. Andrews & G.J. Sanders, London: Chapman & Hall Medical, 1993, 256 pp. £52.50.

The advances made during the last decade in the knowledge of physiology and treatment of radiotherapy and chemotherapy-induced emesis have been so impressive that a comprehensive textbook aimed at compiling the vast amount of information in this field has been long overdue. In this respect, the book by Andrews and Sanger fulfils an important gap in medical literature. The contributions of the editors in the field of antiemetics have been so transcendent that I believe they are among the most authoritative investigators to undertake the enterprise of editing such a book.

The book offers many excellent reviews that I am sure will be highly appreciated by preclinical and clinical investigators involved or interested in antiemetic therapy. I personally enjoyed the updates on measurement and mechanisms of nausea, changes in gastro-intestinal motility associated with emesis, neurotransmitters and receptors of the emetic pathway, mechanisms or emesis induced by chemotherapy and radiotherapy, and pharmacology of antiemetic agents. Many of the chapters include a significant number of suggestions and considerations able to generate interesting ideas to be tested in preclinical and clinical setting. However, no book is perfect and, as a clinician, I am somewhat critical of the selection of certain other chapters. Although the authors stated in the preface that 'the problems of nausea and vomiting are set in a more general context of anti-cancer therapy by a detailed study of the pharmacokinetics and pharmacodinamics of chemotherapeutic drugs and an examination of their general toxicities', I do not find that this argument justifies the inclusion of two chapters on such general topics as 'The pharmacokinetics and pharmacodynamics of chemotherapeutic agents' and 'Toxicology of chemotherapeutic agents' in a book specifically devoted to emetic mechanisms and antiemetic therapy. In addition, despite its title, the book devotes just one brief chapter out of eleven to the practical management of chemotherapy-induced emesis. In my opinion, those readers involved in the clinical treatment of cancer patients would have appreciated a more detailed analysis of the plethora of problems associated with the management of nausea and vomiting in the clinical setting. In spite of my reservations concerning some of the selections, I feel that this book constitutes an excellent and necessary reference text which should occupy a preferential place in the library of all those involved in one way or another in the physiology and management of cancer treatment-related emesis.

M. Martin